# Changes in Tonic Alertness but Not Voluntary Temporal Preparation Modulate the Attention Elicited by Task-Relevant Gaze and Arrow Cues

**DOI:** 10.3390/vision2020018

**Published:** 2018-04-07

**Authors:** Dana A. Hayward, Jelena Ristic

**Affiliations:** 1 Department of Psychology, University of Alberta, Edmonton, AB T6G 2R3, Canada; 2 Department of Psychology, McGill University, Montreal, QC H3A 1B1, Canada

**Keywords:** spatial attention, temporal attention, attentional orienting, reflexive attention, voluntary attention, social attention, automated symbolic orienting, visual attention

## Abstract

Attention is engaged differently depending on the type and utility of an attentional cue. Some cues like visual transients or social gaze engage attention effortlessly. Others like symbols or geometric shapes require task-relevant deliberate processing. In the laboratory, these effects are often measured using a cuing procedure, which typically manipulates cue type and its utility for the task. Recent research however has uncovered that in addition to spatial orienting, this popular paradigm also engages two additional processes—tonic alertness and voluntary temporal preparation—both of which have been found to modulate spatial orienting elicited by task-irrelevant cues but not task-relevant symbols. Here we assessed whether changes in tonic alertness and voluntary temporal preparation also modulated attentional orienting elicited by task-relevant social gaze and nonsocial arrow cues. Our results indicated that while the effects of spatial attention were reliable in all conditions and did not vary with cue type, the magnitude of orienting was larger under high tonic alertness. Thus, while the cue’s task utility appears to have the power to robustly drive attentional orienting, changes in tonic alertness may modulate the magnitude of such deliberate shifts of attention elicited by task-relevant central social and nonsocial cues.

## 1. Introduction

Spatial attention is engaged differentially depending on the type of attentional cue [1,2,3,4,5]. While some cues like peripheral luminance changes engage attention relatively effortlessly in a reflexive manner, others like centrally presented geometric shapes engage attention in a more voluntary fashion, requiring deliberate effort. Experimentally, these effects are often studied using the Posner cuing task [3,6]. In this task, an attentional cue that indicates a potential target location is presented. Then, after a variable amount of time (i.e., the cue–target interval) a target demanding a response is presented either in the location indicated by the cue (i.e., cued target) or in some other location (i.e., uncued target). If the cue has engaged attention, participants’ performance is facilitated (i.e., is faster and/or more accurate) for cued relative to uncued targets. 

### 1.1. Measuring Attentional Effects

The cue’s ability to engage attention is often studied by manipulating its spatial predictiveness by making it either spatially uninformative or spatially informative about an upcoming target, e.g., [7]. Studies show that cues like visual transients [3,8,9] as well as averted social eye gaze [10,11,12] need not be spatially informative to engage attention. By contrast, more symbolic cues like geometric shapes [4], letters [13] or numbers [1,5,14] require a level of task-relevant spatial information to engage attention. However, in addition to cue type and spatial predictiveness, the cuing task also engages two other processes, namely tonic alertness and voluntary temporal preparation, both of which have recently been found to modulate the observed attentional effects [2,15,16,17,18,19,20]. 

#### 1.1.1. Tonic Alertness

Tonic alertness is understood to reflect long-term changes in participants’ overall readiness to respond [21,22]. Accordingly, its effects have been investigated by manipulating the number of trials that require a response, i.e., the number of trials in which a response target appears e.g., [2,17,18,19]. The inclusion of no-target trials is common, especially in simple detection tasks, as this practice serves to both increase participants’ tonic alertness and to guard against a potential response bias in the case when all trials contain a response target. When the number of no-target trials is relatively low (e.g., 5%), a strong coupling between the cue and target occurs, creating a high level of tonic alertness. This is indexed by both overall facilitated response times (RTs) and the presence of the foreperiod effect, or the speeding up of RTs with the lengthening of the time interval between the cue and target [23,24,25]. However, when the proportion of no-target trials is increased to for instance 25%, the link between the cue and the target is weakened, and participants’ level of tonic alertness or their readiness to respond consequently decreases. Experimentally, this manipulation results in slower responses overall and the elimination of the foreperiod effect [2,17,19].

#### 1.1.2. Voluntary Temporal Preparation

Voluntary temporal preparation is thought to reflect participants’ implicit entrainment to the timing of cue–target events. That is, in a typical cuing task, which uses a so-called ‘aging’ distribution of trials, an equal number of targets are assigned to appear at each cue–target time [2,15,16,17,22]. However, this practice does not result in equating the probability of target appearance at each cue–target interval. Rather, it increases the likelihood that, given its absence at an early cue–target time, the target will appear at a later cue–target time. This is because as each cue–target interval passes, the number of trials assigned to appear at that time point stays constant while the total number of remaining trials decreases. To illustrate, consider an example with four cue–target intervals and a total of 128 trials, with 32 trials assigned to each cue–target time. At the first cue–target interval, the probability of target appearance is 32/128 trials or *p* = 0.25. However, provided no target occurs at this earliest cue–target time, the probability that the target will occur at the next cue–target time increases to 32/96 trials or *p* = 0.33. The probability of a target occurring at the third cue–target time jumps even further to *p* = 0.50 or 32/64 trials, and finally reaches certainty if all trials contain a response target, i.e., 32/32 trials or *p* = 1. One way to correct for this increasing probability of target presence is to utilize a so-called ‘nonaging’ distribution of trials, in which the number of targets presented at each successive cue–target time is halved. To illustrate, consider the same scenario with four cue–target intervals and 128 total trials. Here, to maintain a target occurrence probability of *p* = 0.50 at each cue–target interval, the number of remaining targets is halved for each successive cue–target time. As such, half of all targets (64/128) are assigned to appear at the shortest cue–target interval, half of the remaining ones at the next cue–target time (32/64), half of those at the following cue–target time (16/32), with the final 8/16 targets occurring at the longest cue–target time. Experimentally, voluntary temporal preparation is also indexed by the foreperiod effect [2,17]. While the foreperiod is present when the task employs an aging distribution of trials, it becomes eliminated when the implicit probability of the target’s temporal appearance is equated using a nonaging distribution [15,16,22]. 

### 1.2. Effects of Tonic Alertness and Voluntary Temporal Preparation on Attentional Orienting

Studies that have investigated the role that changing tonic alertness and voluntary temporal preparation have on orienting of spatial attention generally show that these task parameters modulate both spatial orienting and the foreperiod effect. For instance, when attention is engaged with spatially nonpredictive luminance changes, lowering tonic alertness leads to slowed responses and an abolished foreperiod [17,19]. Eliminating voluntary temporal preparation with this cue also results in an abolished foreperiod effect [15,16,17]. However, indicating that tonic alertness and voluntary preparation affect the foreperiod in an interactive fashion, when both processes are reduced jointly the foreperiod effect becomes reduced such that participants become slowest to respond to targets appearing at the longest cue–target time [17]. Furthermore, spatial attention engaged by visual luminance transients is also modulated by both task parameters [17,19]. Spatial orienting remains unchanged under absent voluntary temporal preparation alone [15,17], but becomes reduced when either tonic alertness [19] or both tonic alertness and voluntary temporal preparation are jointly lowered [17]. 

Changing tonic alertness and voluntary temporal preparation have also been found to affect attentional orienting engaged by spatially nonpredictive central eye gaze and arrow cues [2]. While a reduced foreperiod magnitude with the joint lowering of tonic alertness and voluntary temporal preparation also held for central cues, modulations of attentional effects diverged with cue type. In Hayward and Ristic’s [2] study, social orienting remained generally unaffected by changes in the task parameters except that its magnitude decreased at the longest cue–target time of 925ms when both tonic alertness and voluntary temporal preparation were reduced. Automated orienting on the other hand, which is engaged by spatially nonpredictive arrows [26,27], increased in magnitude under absent voluntary temporal preparation, and was delayed in its onset to the longest 925 ms cue–target interval when the contribution of both processes was reduced. 

Recently, Laidlaw and Kingstone [18] investigated how lowering tonic alertness by decreasing the number of no-target trials from 0% (i.e., 100% of trials contained a target) to 25% (i.e., 75% of trials contained a target) affected automated and voluntary orienting. A typical cuing task with an aging distribution of trials was used, in which either a central task-irrelevant arrow, which did not predict the location of an upcoming target (i.e., engaging automated attention), or a central task-relevant letter (M or W), which correctly indicated the location of an upcoming target in 80% of trials (i.e., engaging voluntary attention) served as attentional cues. The data indicated that whereas automated orienting was abolished under low tonic alertness, voluntary orienting remained robust in both alertness conditions.

### 1.3. The Present Study

In the present investigation, we sought to examine whether systematic changes in tonic alertness and voluntary temporal preparation modulated orienting elicited by central directional task-relevant spatially predictive gaze and arrow cues. Extending Laidlaw and Kingstone [18], the present study investigated the effects of both individual and joint contributions of tonic alertness and voluntary temporal preparation on spatial orienting elicited by central eye gaze and arrow cues. Extending Hayward and Ristic [2], the present study investigated how changing the task parameters influenced spatial orienting when it was engaged by task-relevant gaze and arrow cues. Based on this past work, we expected the foreperiod to be affected by both individual and joint manipulations of tonic alertness and voluntary temporal preparation and to find little influence of changing tonic alertness conditions on voluntary attention.

## 2. Materials and Methods

### 2.1. Participants

A total of 92 participants completed the study (83 females, 9 males; average age: 20.7 years; Standard Deviation (SD) = 3.8). Each person was randomly assigned to receive either the gaze (*n* = 50) or arrow (*n* = 42) cue and either the high (*n* = 44; gaze *n* = 25; arrow *n* = 19) or low (*n* = 48, gaze *n* = 25; arrow *n* = 23) tonic alertness condition. These sample sizes reflect past studies well, which reported medium to large overall cuing effect sizes (*Cohen d*’s between 0.35 and 0.84) using samples ranging from 14 to 48 participants [12,28,29,30]. We did not include or analyze the data from any participants who did not comply with task instructions (i.e., generating response errors on more than 20% of trials). All procedures were in accordance with the Declaration of Helsinki (2008) and were approved by the McGill University Behavioral Research Ethics Board (#81-0909). 

### 2.2. Apparatus and Stimuli

**Figure 1:**
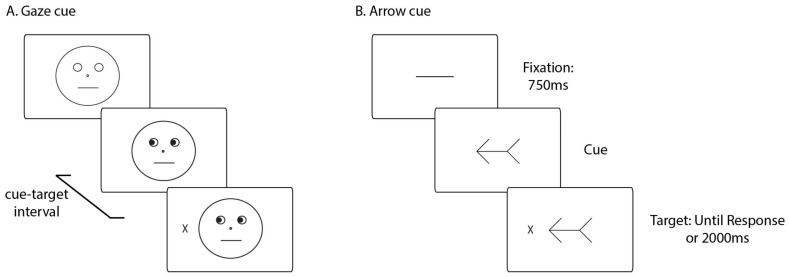
Stimuli and example task sequence for gaze (1A) and arrow (1B) cues. All trials started with a fixation screen, displaying either a blank face or a straight line for 750 ms. Then, the central gaze or arrow cue, indicating either the left or right location was shown. After the variable cue–target time, the response target (a capital letter X) was presented on the left or right of the fixation. The cue and the target remained on the screen until response or until 2000 ms. Cue direction indicated the correct location of the target in 75% of trials. Note: Stimuli are not drawn to scale.

The stimuli and experimental setup replicate our previous work [2]. Black and white line drawings of a schematic face (Figure 1A) and an arrow (Figure 1B) served as cues. The face consisted of a face outline (9.4°), pupils (i.e., black filled-in circles centered within eye outlines; 0.7°), mouth (3°), and a nose (0.3°). The arrow consisted of a horizontal line (4.6°) with an arrowhead and an arrow tail (each 1.9°). A capital letter ‘X’ (1°) served as the response target, appearing with an eccentricity of 6.4° to the left or right of central fixation. The stimuli were shown on a 16-in cathode ray tube (CRT) monitor at an approximate distance of 57 cm.

### 2.3. Design

The design also followed from Hayward and Ristic [2], except that the attentional cues were spatially predictive of the target location. The cue’s direction indicated the correct location of an upcoming target in 75% of trials. Cue type (gaze vs. arrow) and tonic alertness (high vs. low) were manipulated between subjects. Voluntary temporal preparation (present vs. absent), cue validity (cued vs. uncued), and cue–target interval were manipulated within subjects. Voluntary temporal preparation was blocked and presented in a random order between participants. Cue validity and cue–target interval were intermixed and presented in a pseudorandom order within participants. On any given trial, gaze and arrow cues indicated either a left or right spatial location, and the target appeared on either the left or right side. 

Tonic alertness was manipulated by changing the number of no-target trials. While a cue appeared in each trial, the target appeared with different occurrence certainty across the two tonic alertness conditions. For high tonic alertness, a target was presented in 94% of the trials and no target was shown in 6% of the trials. In contrast, for low tonic alertness, a target was presented in 75% of the trials and no target was shown in 25% of the trials. Note that Laidlaw and Kingstone [18] used the term *temporal attention* to refer to their manipulation of changing the number of no-target trials. Here we use the more general term *tonic alertness* to refer to the same manipulation in order to both maintain continuity with the existing literature e.g., [2,15,16,17,20,22,31] and to individuate this factor from the factor of voluntary temporal preparation, which has been well documented to lead to changes in the implicit orienting of temporal attention, e.g., [24,32,33,34].

Voluntary temporal preparation was manipulated by altering the distribution of trials across the cue–target intervals from an aging to a nonaging one, e.g., [2,15,16,17,22,31]. Voluntary temporal preparation was present when the aging distribution of trials was used. This distribution presents the same number of trials at each cue–target interval but leads to an increased likelihood of target appearance with the lengthening of the cue–target time. Voluntary temporal preparation was absent when the nonaging distribution of trials was used. This distribution presents a different number of trials at each cue–target interval but results in an equal likelihood of target appearance across all cue–target times, e.g., [2,17]. In order to equate the probability of target occurrence across all cue–target times for the nonaging distribution and to also manipulate the percentage of no-target trials, the total number of cue–target intervals differed for the high and low tonic alertness conditions, see [2,15,17] for a thorough explanation. To preserve high tonic alertness and the nonaging distribution, four cue–target intervals are required. To preserve low tonic alertness and the nonaging distribution, two cue–target intervals are required. The number of cue–target intervals and trial counts for all experimental conditions are shown in Table 1. Note that due to an increased number of cue–target times, the high tonic alertness conditions contained more test trials (average of 1056) compared to the low tonic alertness conditions (average of 876). However, it is unlikely that this variable influenced our results by creating differential fatigue effects for two reasons. One, the total testing time did not differ much across the two tonic alertness conditions (about 31 min vs. 31.8 min, not including individual RTs, for the high vs. the low tonic alertness, respectively). Two, an analysis of overall RTs as a function of testing block (1–4) and tonic alertness (high vs. low) also indicated no reliable differences in the fluctuations of responses across the high and low tonic alertness conditions [*Testing block* × *Tonic alertness*; *F*(3,264) = 0.61, *p* = 0.6]. Future work in which fatigue effects are manipulated and measured directly is needed to examine whether this factor plays a role in the reported performance differences. 

### 2.4. Procedure

Each trial began with the presentation of either a blank face or a straight line for 750 ms. Then, pupils looking left or right or an arrow pointing left or right appeared. After the variable cue–target time, the target was presented on the left or right side of fixation. Participants were instructed to press the spacebar quickly and accurately once they detected its onset, and to withhold a response if no target appeared. They were also instructed that the cue’s direction indicated the correct target location in 75% of trials and to maintain central fixation throughout the task. In order to ensure that participants utilized the cue–target timing information implicitly, as in past work [2,15,16,17], they were not given any instructions about the cue–target temporal links. The cue and the target remained visible until response or until 2000 ms. RT was measured from target onset. The task was divided into four blocks of trials, with five practice trials run at the start.

## 3. Results

Overall the task was performed well. Anticipations (RT < 100 ms), timeouts (RT > 1000 ms) and false alarms (i.e., responding on a no-target trial) accounted for less than 2% of trials in any condition. Mean correct RTs were examined at the common cue–target intervals of 100 and 925 ms, as this was the only way to fully and directly compare modulations in spatial orienting as a function of all experimental conditions, i.e., isolated and combined influences of tonic alertness and voluntary temporal preparation [2,17]. 

**Figure 2:**
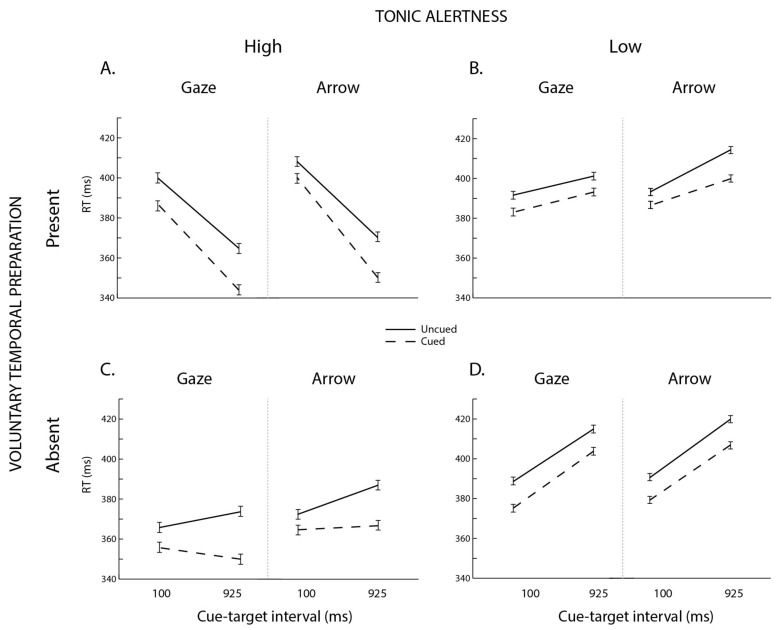
Results. Mean interparticipant correct Response Times (RTs) as a function of tonic alertness (high; low), voluntary temporal preparation (present; absent), cue type (gaze; arrow), cue validity (cued; uncued), and cue–target time (100 ms; 925 ms). Error bars represent the standard error of the difference between the means.

We first examined the data using an omnibus mixed effects Analysis of Variance (ANOVA), with cue type (gaze vs. arrow) and tonic alertness (high vs. low) included as between-subjects variables and voluntary temporal preparation (present vs. absent), cue validity (cued vs. uncued), and cue–target interval (100 and 925 ms) included as within-subject variables. Follow-up analyses were conducted at each cue–target interval separately in order to connect with Laidlaw and Kingstone’s [18] work. Figure 2 illustrates mean interparticipant correct RTs as a function of tonic alertness, voluntary temporal preparation, cue type, cue validity, and cue–target time. 

### 3.1. Omnibus ANOVA

The omnibus ANOVA returned reliable main effects for *tonic alertness* [*F*(1,88) = 6.2, *p* = 0.015, η_p_^2^ = 0.07 and *cue validity* [*F*(1,88) = 151.4, *p* < 0.0001, η_p_^2^ = 0.63], with responses overall faster for the high relative to the low tonic alertness condition, and for cued relative to uncued trials. As expected and as illustrated in Figure 2, the foreperiod was also modulated by changes in tonic alertness and voluntary temporal preparation. This observation was supported by reliable interactions arising between *tonic alertness*, *voluntary temporal preparation*, and *cue–target interval*. That is, and replicating past reports [2,17], the foreperiod effect was reduced by both lowering tonic alertness [Figure 2B; *cue–target interval* × *tonic alertness*, *F*(1,88) = 64.1, *p* < 0.0001, η_p_^2^ = 0.42] and voluntary temporal preparation individually [Figure 2C; *cue–target interval* × *voluntary temporal preparation*, *F*(1,88) = 144.9, *p* < 0.0001, η_p_^2^ = 0.62], as well as by decreasing both processes in conjunction [Figure 2D; *voluntary temporal preparation* × *tonic alertness*, *F*(1,88) = 5.3, *p* = 0.024, η_p_^2^ = 0.06; *voluntary temporal preparation* × *tonic alertness* × *cue–target interval*; *F*(1,88) = 39.9, *p* < 0.0001, η_p_^2^ = 0.31]. 

The ANOVA also indicated that the magnitude of spatial orienting was modulated by the manipulations of tonic alertness and voluntary temporal preparation. First, a two-way interaction between *cue validity* and *cue–target interval* [*F*(1,88) = 15.4, *p* = 0.0002, η_p_^2^ = 0.15] indicated that, as is customary for voluntary attention [29,35], the magnitude of attentional orienting (i.e., the RT difference between cued and uncued targets) grew overall with increasing cue–target time. However, this finding held only for the high alertness condition as confirmed by a reliable *cue validity* by *tonic alertness* interaction [*F*(1,88) = 4.7, *p* = 0.032, η_p_^2^ = 0.05], showing that the magnitude of orienting was larger under high (Figure 2A,C) relative to low (Figure 2B,D) tonic alertness. Finally, a three-way interaction between *cue validity*, *tonic alertness*, and *cue–target interval* [*F*(1,88) = 8.6, *p* = 0.0043, η_p_^2^ = 0.09] indicated that the effect of high tonic alertness on spatial orienting was more pronounced at the longer cue–target time, as the magnitude of spatial orienting increased at the 925 ms cue-target interval for the high but not the low tonic alertness condition. No other effects or interactions were reliable, including any involving *cue type* [*cue type* × *voluntary temporal preparation* × *tonic alertness* × *cue–target interval*, *F*(1,88) = 3.5, *p* = 0.063, η_p_^2^ = 0.04; all other *Fs* < 3, *ps* > 0.1].

Thus, when we assessed the influence of tonic alertness and voluntary temporal preparation on spatial orienting elicited by task-relevant central gaze and arrow cues, we found that the magnitude of attentional orienting, i.e., the RT difference between cued and uncued trials, became larger under high relative to low tonic alertness at the longest cue–target time. Our data also indicated that this result did not vary as a function of cue type, with the results remaining steady across social gaze and nonsocial arrow cues. 

### 3.2. Follow-Up Analyses

We next analyzed the data separately for the condition in which tonic alertness was low and voluntary temporal preparation was present, which mirrors Laidlaw and Kingstone’s [18] low alertness condition, and the condition in which tonic alertness was low and voluntary temporal preparation was absent, which was employed in the present study e.g., [2]. As a reminder, Laidlaw and Kingstone [18] found that voluntary attentional orienting elicited by spatially predictive central letter cues was unaffected by lowered tonic alertness, remaining reliable only at the longer cue–target intervals. 

We used four separate one-way ANOVAs to examine the difference between cued and uncued RTs in the two task conditions (low tonic alertness and high voluntary temporal preparation; low alertness and low voluntary temporal preparation) for each cue–target time (100 ms; 925 ms). In the low tonic alertness and present voluntary temporal preparation case, robust main effects of *cue validity* were found at both the early [100 ms, *F*(1,47) = 17.6, *p* < 0.0001, η_p_^2^ = 0.27] and the late [925 ms, *F*(1,47) = 24.0, *p* < 0.0001, η_p_^2^ = 0.34] cue–target intervals. The same result emerged in the condition in which both tonic alertness and voluntary temporal preparation were absent, with reliable main effects of *cue validity* at both the early [100 ms; *F*(1,47) = 50.4, *p* < 0.0001, η_p_^2^ = 0.52] and the late [925 ms; *F*(1,47) = 13.7, *p* = 0.001, η_p_^2^ = 0.23] cue–target intervals. As such, these data indicated robust orienting effects at both early and late cue–target times, when tonic alertness was manipulated jointly with voluntary temporal preparation and when it was manipulated alone. We return to this point in the discussion.

Taken together, the results from our study showed that when directional gaze and arrow cues were made spatially relevant, attentional orienting remained robust across changing manipulations of tonic alertness and voluntary temporal preparation, but showed an increased magnitude of orienting at the longer cue–target time in the high alertness condition. No differences between the two cue types emerged.

## 4. Discussion

Motivated by recent work [2,17,18] indicating the sensitivity of attentional effects to cue types, task settings, and/or task-relevance, here we sought to determine whether changes in the cuing task parameters of tonic alertness and voluntary temporal preparation modulated spatial orienting elicited by task-relevant central directional gaze and arrow cues. We manipulated tonic alertness by changing the percentage of no-target trials from 6% in the high tonic alertness condition to 25% in the low tonic alertness condition. We manipulated voluntary temporal preparation by presenting the trial sequences using either an aging or a nonaging distribution, which respectively serve to preserve or eliminate the effects of voluntary temporal preparation. The two factors were manipulated in isolation and jointly. Replicating past studies [2,15,16,17,18,19,22], we found that lowering tonic alertness and voluntary temporal preparation both in isolation and in conjunction reduced the foreperiod effect. In contrast, while attentional orienting was robust across all conditions, we observed the typical voluntary pattern of orienting with an increasing magnitude of attentional effects at longer cue–target intervals in the high tonic alertness condition only. Finally, we found that attentional effects did not change as a function of cue type, remaining steady across social gaze and nonsocial arrow cues. 

On first glance these data appear to contrast Laidlaw and Kingstone [18], who did not find modulations of voluntary attention with changes in tonic alertness. There are at least two points to consider here. One is that the task conditions between these two studies did not fully match, which makes direct comparisons across the two studies difficult. More specifically, while Laidlaw and Kingstone [18] manipulated tonic alertness by lowering the percentage of no-target trials from 0 to 25%, the present study manipulated tonic alertness by lowering the percentage of no-target trials from 6 to 25%. As such, Laidlaw and Kingstone’s [18] high alertness condition preserved the target’s temporal uncertainty but eliminated its occurrence uncertainty, e.g., [22], while our design preserved both the temporal and occurrence uncertainty, allowing a more equal comparison between high and low alertness conditions.

Two, the designs also diverged based on cue type. Laidlaw and Kingstone [18] studied the effects of changing cuing task parameters on voluntary orienting elicited by a nondirectional symbolic letter cue. Here, we studied the effects of changing task parameters on orienting elicited by task-relevant directional gaze and arrow cues. Unlike nondirectional cues like letters or geometric shapes, when manipulated as task-irrelevant or spatially nonpredictive, directional gaze and arrows normally elicit orienting effects at both early and late cue–target times, e.g., [11,36,37]. Furthermore, when they are made task-relevant or spatially predictive, arrow cues have been found to produce large, so-called combined effects, which occur robustly at both early and late cue–target times and are proposed to reflect a combination of reflexive and voluntary orienting [4,26,27,29,38]. Voluntary orienting elicited by central symbolic cues on the other hand is thought to elicit a more pure form of voluntary attention, with effortful attentional effects beginning to emerge only at mid cue–target intervals of about 300–500 ms [13]. It is therefore possible that while our data show effects of changing tonic alertness on attentional orienting elicited by gaze and arrow cues, these results may reflect the differential cue-specific effects that are normally exerted on spatial attention by social, automated, and symbolic cues. Thus, although overall the present results appear to diverge from Laidlaw and Kingstone [18], these two studies conceptually converge to show that the typical cue-specific attention effects elicited by task-relevant cues generally remain robust with changes in cuing task settings, but that those task parameters may also modulate the magnitude of attentional orienting elicited by different types of cues in a divergent fashion. 

The present study also extends our previous work with spatially nonpredictive directional cues [2,17,32]. In those studies, we showed that spatial attention varied with both task parameters and cue type. While social orienting elicited by spatially nonpredictive gaze cues was mostly resilient to changing task parameters, automated orienting elicited by spatially nonpredictive arrows was affected by changes in both tonic alertness and voluntary temporal preparation. Specifically, while automated orienting appears to be accelerated by the presence of implicit temporal predictability between the cue–target events (i.e., voluntary temporal preparation) it remains unaffected when that temporal information is manipulated explicitly [32]. However, showing diverging effects across social and nonsocial cues, lowering both tonic alertness and voluntary temporal preparation resulted in a delayed onset of automated but not social orienting [2]. The present results further show that when directional central cues are made task-relevant, or spatially informative of a target, this task setting appears to be able to dominate the cue-specific effects that emerge when those same cues are made spatially nonpredictive. That is, here we found that when spatially predictive, both gaze and arrow cues produced attentional orienting effects that did not vary as a function of cue type. As such, the present results suggest that the spatial predictiveness of a cue may be a powerful modulator of task performance over and above any influences of a particular cue type. 

Finally, and similarly to our previous work [2,17], the present data also revealed that the typical foreperiod was present only when both tonic alertness and voluntary temporal preparation were present. Experimentally lowering either or both processes contributed to a reduction in the foreperiod magnitude. We have argued previously [2] that the decrease in the foreperiod magnitude may reflect a reduction in task-related target expectancy, as when both processes are reduced the target is both less likely to appear and the task sequence provides no implicit temporal information about when it would appear. The present work additionally indicates that the pattern of overall slowing of RTs at later cue–target times occurs similarly regardless of whether the cue is spatially predictive or spatially nonpredictive, indicating a more important role of the task’s temporal rather than spatial parameters in the foreperiod effect. 

In sum, the present study once again demonstrated that the cuing task settings modulate the observed spatial attentional effects. While our past work showed that different task-irrelevant cue types have the power to drive attention differentially, the present work shows that when those cues are made relevant for the task their intrinsic cue effects may become superseded by the task parameters. 

## Figures and Tables

**Table 1:** Trial counts for all experimental conditions.

	Target Present								No Target
Cue–target time	100 ms		375 ms		650 ms		925 ms		
	cued	uncued	cued	uncued	cued	uncued	cued	uncued	
High Alertness									
Aging	192	64	192	64	192	64	192	64	64
Nonaging	384	128	192	64	96	32	48	16	64
Low Alertness									
Aging	240	80	--	--	--	--	240	80	216
Nonaging	336	112	--	--	--	--	168	56	224
